# Supplementary material for: Palliative care follow-up for cancer patients combining day-hospital visits and telemedicine: What feasibility?
Source: PLoS One. 2026 Mar 24;21(3):e0318050. doi: 10.1371/journal.pone.0318050 (PMC13012492; doi:10.1371/journal.pone.0318050)
Supplement: S1 File — (DOCX) [file pone.0318050.s001.docx]

S1 File : **Questionnaire**

**Patient anonymization number:** ………

**Telemsos Study**
Contribution of telemedicine to the follow-up of patients with chronic cancer disease by the mobile supportive care team.

**Dear Sir or Madam,**
You have agreed to participate in the Telemsos study, which aims to generate information to help us better adapt our healthcare services (telemedicine) for patients followed by the mobile supportive care team.
Please find below Questionnaire No. 1, which will help us get to know you better (epidemiological data) and evaluate, among other things, your expectations or concerns regarding telemedicine consultations. Completing it will take approximately 15 minutes.
This questionnaire is anonymous and will be stored securely.

Thank you for your participation.

For any questions regarding this questionnaire, please contact us at: [**couderc.bettina@iuct-oncopole.fr**](mailto:couderc.bettina@iuct-oncopole.fr)

## **Section 1: Socio-demographic / Epidemiological Characteristics**

1. **I am:**
   ☐ Male
   ☐ Female
2. **Age:** ____ years
3. **What is the highest level of education you have completed?**
   ☐ No diploma
   ☐ Basic Education Certificate (CFG), Primary School Certificate, Lower Secondary Diploma (BEPC)
   ☐ Vocational Training Certificate (CAP), Vocational Studies Certificate (BEP)
   ☐ Baccalaureate or equivalent diploma
   ☐ 1–2 years of higher education
   ☐ 3 years of higher education or more
   ☐ Other (please specify): _____________
4. **What is your current or, if retired, your most recent occupational category?**
   ☐ Farmer or spouse working on the farm
   ☐ Artisan, shopkeeper, business owner, or collaborating spouse
   ☐ Executive, higher intellectual profession (engineer, physician, etc.)
   ☐ Intermediate profession (primary school teacher, nurse, social worker, technician, supervisor, etc.)
   ☐ Employee (office or retail employee, childcare worker, service agent, etc.)
   ☐ Manual worker
   ☐ Homemaker, no occupation
   ☐ Other (please specify): _____________
5. **Do you need someone to help you understand prescriptions or medical documents provided by your doctor or pharmacist?**
   ☐ Yes
   ☐ No
6. **At home, do you speak a language other than French?**
   ☐ Yes (please specify): __________
   ☐ No

## **Section 2: Place of Residence**

1. **How far is your home from IUCT-Oncopole?** ____ km
2. **How much time does it take you to travel there for a consultation?** ____ minutes
3. **Do you live with a partner?**
   ☐ Yes
   ☐ No
4. **Who else lives under the same roof? (check all that apply)**
   ☐ Your children
   ☐ Your parents
   ☐ Other relatives (uncle, aunt, brother, sister)
   ☐ Housemates/co-tenants
5. **If you live with a partner and/or others, do you consider them your caregivers?**
   ☐ Yes
   ☐ No

**If yes, you consider that they help you:**
☐ A little
☐ A lot
☐ They are essential for daily life activities

## **Section 3: Digital Access and Use**

1. **Do you have Internet access?**
   ☐ No, I do not have Internet access
   ☐ Yes, on my mobile phone
   ☐ Yes, on a PC/tablet (via Wi-Fi)
   ☐ Yes, on both my mobile phone and a PC/tablet
2. **How often do you use a computer or phone screen?**
   ☐ I never use screens
   ☐ At least once a day
   ☐ At least once a week
   ☐ At least once a month
3. **Have you ever had a consultation with a doctor or healthcare professional by telephone from your home?**
   ☐ Never
   ☐ A few times
   ☐ Often
   ☐ Very often
4. **Have you ever had a consultation with a doctor or healthcare professional via telemedicine (computer + camera) from your home?**
   ☐ Never
   ☐ A few times
   ☐ Often
   ☐ Very often

## **Section 4: Opinions on Telemedicine**

**For the following statements, please indicate your level of agreement:**
(1 = Strongly disagree, 2 = Somewhat disagree, 3 = Neutral/No opinion, 4 = Somewhat agree, 5 = Strongly agree)

### ****Regarding your care:****

1. Telemedicine is not suitable for my medical follow-up. [1–5]
2. Telemedicine could be useful to me, but only for certain medical procedures. [1–5]
3. Telemedicine spares me from traveling to the hospital for my medical questions. [1–5]
4. Telemedicine allows me to save time. [1–5]
5. I feel reassured being able to consult my doctor without the risk of being infected or becoming fatigued. [1–5]

### ****Regarding the process:****

1. I am afraid I will not understand how to connect. [1–5]
2. I am afraid I will not hear/understand what the doctor says. [1–5]
3. I would prefer to have someone help me connect. [1–5]

### ****Regarding the relationship with the healthcare team:****

1. Telemedicine dehumanizes medicine. [1–5]
2. Telemedicine weakens relationships with caregivers. [1–5]
3. Monthly telemedicine helps maintain a strong connection with the care team. [1–5]
4. Having a screen in between allows me to ask certain questions with less embarrassment. [1–5]
5. Having a screen in between makes it harder for me to express or show certain difficulties. [1–5]
6. A telemedicine consultation is as beneficial as an in-person consultation. [1–5]

### ****Regarding your personal information:****

1. Telemedicine does not ensure the confidentiality of my conversations with the care team. [1–5]
2. I am afraid that people other than my doctor might listen to my consultation. [1–5]
3. I do not know if consultations are recorded and stored somewhere. [1–5]

### ****Regarding the involvement of your relatives (partner, children, parents, caregiver):****

1. Telemedicine makes it easier for my relatives to accompany me compared to a day-hospital visit. [1–5]
2. I feel uncomfortable with the idea that people living under the same roof might hear my consultation. [1–5]
