# Supplementary material for: Palliative care follow-up for cancer patients combining day-hospital visits and telemedicine: What feasibility?
Source: PLoS One. 2026 Mar 24;21(3):e0318050. doi: 10.1371/journal.pone.0318050 (PMC13012492; doi:10.1371/journal.pone.0318050)
Supplement: S2 File — (DOCX) [file pone.0318050.s002.docx]

## **S2 File – Semi-Structured Interview Protocol**

**Study:** Telemsos – Contribution of telemedicine to the follow-up of patients with chronic cancer disease
**Purpose:** To explore patients’ experiences and perceptions of teleconsultations with the mobile supportive care team (IUCT-Oncopole).

### ****Introduction Script (to be read to participants):****

Hello,
Thank you very much for agreeing to participate in this semi-structured interview regarding your experience with teleconsultations provided by the supportive care team at IUCT-Oncopole.

The purpose of this interview is to gather your feedback and feelings about teleconsultations. You are free not to answer any questions and/or to stop the interview at any time.

All your personal data will be anonymized to ensure complete confidentiality and freedom of expression.

This is an open discussion based on a series of topics. The interview will be recorded and transcribed for analysis, and all data will be stored securely.

### ****Interview Questions and Prompts:****

**1. First contact with the supportive care team**

- **Main question:** Could you tell me how you first met the mobile supportive care team at Oncopole?
- **Prompts:**
  - At what stage in your care pathway?
  - Who referred you (doctor/service)?
- **Notes:** ____________________________________________

**2. Today’s in-person consultation**

- **Main question:** Can you describe your in-person consultation today?
- **Prompts:**
  - Were you accompanied?
  - How was the trip organized?
  - Distance traveled?
- **Notes:** ____________________________________________

**3. Care proposed/prescribed today**

- **Main question:** What treatments or care were proposed or prescribed during this consultation?
- **Notes:** ____________________________________________

**4. Interaction with the team**

- **Main question:** How did your exchanges with the team go?
- **Prompts:**
  - Which professionals did you meet?
  - Did you understand the care provided?
  - Were you satisfied with the care?
- **Notes:** ____________________________________________

**5. After the first in-person consultation**

- **Main question:** Following the care prescribed during the first in-person visit, how did things go at home?
- **Prompts:**
  - Did you understand your treatments?
  - How was home care organized?
  - Did someone assist you?
- **Notes:** ____________________________________________

**6. Organization of the first teleconsultation**

- **Main question:** How was your first teleconsultation organized?
- **Prompts:**
  - Who scheduled it?
  - How were availabilities coordinated?
  - How long after the in-person visit? Did you find this interval long or short?
- **Notes:** ____________________________________________

**7. First teleconsultation experience**

- **Main question:** Can you describe how your first teleconsultation went?
- **Prompts:**
  - Where in your home?
  - Time of day?
  - Who was present?
  - How did you prepare?
- **Notes:** ____________________________________________

**8. Purpose of the teleconsultation**

- **Main question:** What was the reason for this teleconsultation?
- **Prompts:**
  - With which healthcare professional?
  - What symptoms/issues were addressed?
- **Notes:** ____________________________________________

**9. Expectations and apprehensions before teleconsultation**

- **Main question:** Did you have any concerns before your teleconsultation?
- **Prompts:** About what? (connection, communication, information, etc.)
- **Notes:** ____________________________________________

**10. Comfort during teleconsultation**

- **Main question:** During the teleconsultation, did you feel comfortable or uncomfortable?
- **Prompts:** In relation to what? (connection, exchanges, information, etc.)
- **Notes:** ____________________________________________

**11. Difficulties encountered**

- **Main question:** Did you encounter any difficulties during the teleconsultation?
- **Prompts:**
  - Technical problems?
  - Difficulty expressing yourself?
  - Difficulty understanding the healthcare professional?
- **Notes:** ____________________________________________

**12. Support from relatives**

- **Main question:** Did any relatives or friends help you with the teleconsultation?
- **Prompts:**
  - Before, during, or after?
  - Who?
  - In what way (connection, explaining, remembering information)?
- **Notes:** ____________________________________________

**13. Subsequent teleconsultations**

- **Main question:** Have you had any other teleconsultations after the first?
- **Prompts:**
  - For what reasons?
  - At whose request?
- **Notes:** ____________________________________________

**14. Comparison of subsequent teleconsultations**

- **Main question:** How did these subsequent teleconsultations compare to the first one?
- **Notes:** ____________________________________________

**15. Overall impressions**

- **Main question:** Could you share your general impression of all the teleconsultations you have had?
- **Prompts:**
  - Differences between teleconsultations?
  - Compared to in-person consultations?
- **Notes:** ____________________________________________

### ****Closing Script:****

Thank you for sharing your experience and taking the time to participate. Your feedback will greatly help us improve supportive care and telemedicine services for patients.
