# Supplementary material for: Palliative care follow-up for cancer patients combining day-hospital visits and telemedicine: What feasibility?
Source: PLoS One. 2026 Mar 24;21(3):e0318050. doi: 10.1371/journal.pone.0318050 (PMC13012492; doi:10.1371/journal.pone.0318050)
Supplement: Attachment — Submitted filename: Response to Reviewers n.docx [file pone.0318050.s004.docx]

Response to Reviewers

Dear Editor,

Please find attached our point-by-point response to the reviewers, along with the revised version of the manuscript, in which all the requested revisions have been addressed. We sincerely thank the reviewers for their insightful comments and suggestions, which have significantly improved the quality of our manuscript. We hope that the revised version now meets their expectations.

As the manuscript has been reviewed and corrected by a native English speaker, a substantial number of modifications have been made. To facilitate the reviewers' verification of the most critical changes, we are submitting both a version with tracked changes and a clean revised version.

We remain available for any further modifications if required.

Thank you for your time and consideration.

Best regards,
Bettina Couderc

**Reviewers' Comments and Responses**

Reviewer #1: The paper presents an interesting and relevant topic. However, it suffers from several drawbacks that need to be corrected before its possible acceptance.

- The abstract needs to be rewritten, especially clarify the background, after objectives.

We have rewritten the abstract to better align it with the manuscript, as suggested by the reviewer.

Abstract:

Background: Advances in cancer treatments have increased life expectancy, leading to a growing need for palliative care services. Due to the saturation of hospital facilities, teleconsultation is emerging as an innovative solution to improve patient follow-up. This study aims to assess the acceptability and feasibility of monthly teleconsultations for chronically ill cancer patients newly referred to palliative care. Methods: Thirty-six patients were included based on predefined criteria, specifically ownership of a connected digital device and a life expectancy of at least three months. The study design consisted of an initial in-person palliative care consultation, followed by two monthly teleconsultations and a three-month follow-up assessment. Data collection included quantitative analysis from patient-reported questionnaires and qualitative insights from semi-structured interviews. Results: The findings revealed high acceptability, with 100% of recruited patients agreeing to participate in teleconsultation, citing benefits such as more frequent follow-ups and reduced travel-related fatigue. Notably, adherence to teleconsultation was independent of demographic factors, including age, gender, or socioeconomic status. Observed benefits included improved symptom management, enhanced overall quality of care, stronger patient-provider relationships, and a reduction in emergency hospitalizations. However, challenges were identified, including that 64% of patients died within three months, indicating late referral to palliative care, and 5% lacked digital access, highlighting healthcare disparities; additionally, some patients required assistance with technology. Conclusions: Teleconsultation serves as a valuable complementary tool in palliative oncology care, enhancing care continuity and reducing unplanned hospital admissions. Nonetheless, this study underscores the necessity for earlier integration of palliative care and targeted efforts to address digital access inequalities.

- I advise the authors to professionally proofread their manuscript prior to resubmitting.

The manuscript was written by a multidisciplinary team of professionals (physicians, sociologists, ethicists) and reviewed by all authors. Additionally, it has been proofread by a native English speaker.

- Introduction is complet in the health area. However, I think it could better frame the problem under study, indicating more explicitly the factors that were at the basis of the research.
We have added contextual elements to the introduction, and some aspects have also been incorporated at the beginning of the discussion. The key factors underlying our research are:

- An increasing number of patients with advanced-stage cancer but a life expectancy of more than six months will be referred to palliative care departments in the coming years. Advances in cancer treatments allow many patients to transition into a chronic disease phase, requiring follow-up for months or even years without a curative treatment plan. French hospitals are currently considering the implementation of a structured follow-up for these patients, initially in collaboration with oncologists and subsequently under the exclusive care of palliative care teams. The current trend favors the early involvement of palliative care physicians, particularly in gynecologic oncology, to ensure continuity of care when curative treatments are discontinued. Additionally, there is a growing emphasis on deprescription to improve the quality of life in end-of-life patients and to avoid aggressive treatments in the last two months of life, in accordance with ASCO recommendations. These factors will inevitably increase the workload of palliative care teams in the medium term.

- Due to limited human resources and overloaded healthcare structures, hospitals currently offer only quarterly follow-ups for patients exclusively under palliative care. However, rapid health deterioration can occur between two in-person consultations (every three months), often leading to emergency hospitalizations or intensive care admissions, where maximalist interventions are systematically applied. This approach is not consistent with palliative care policies that prioritize quality of life and discourage aggressive treatments in the final month of life.

- It would be beneficial to anticipate these sudden health deteriorations. A monthly follow-up consultation could be a viable solution.

- Patients under palliative care follow-up could benefit from teleconsultations (video consultations).

- Our study aims to assess the acceptability and feasibility of a monthly teleconsultation follow-up for oncology patients, without prior selection. The objective is to determine whether a specific patient profile benefits more from teleconsultations or whether all patients could derive advantages from monthly teleconsultations.

- Methods chapter needs more specification of empirical work. For example, I would like to see a better characterization of the target population and sample in the empirical part

We have added Table 2, which presents the characteristics of each patient individually. For example, we have recorded the patients' primary pathology (prior to referral to the palliative care department), their age at inclusion, the number of teleconsultations performed, their potential participation in a clinical trial before referral, their educational and professional background, whether they live alone, with a partner, or with a parent, their prior experience with teleconsultation, and their place of residence (distance from the healthcare facility).

In structural terms, I suggest that the discussion should be combined with the results and that the conclusions should be in a “CONCLUSIONS” chapter.

The results and discussion have been merged in this revised version of the manuscript, while the conclusion remains separate.

Reviewer #2: The study identifies a predominance of affluent female patients, likely influenced by the institute’s focus on breast cancer. However, it does not adequately explore the implications of this demographic concentration. Provide detailed information on recruitment strategies. Specify whether efforts were made to include a broader demographic, such as patients from varied socioeconomic backgrounds, men, or those with other cancer types. Explicitly discuss how this sample demographic may limit the generalizability of findings, particularly for less affluent, male, or geographically isolated populations. If data permits, include subgroup comparisons (e.g., by gender, socioeconomic status, or geography) to identify variations in teleconsultation experiences or outcomes.

The hospital center where the study was conducted does not exclusively manage patients with breast cancer or, more broadly, gynecological cancers (ovarian, endometrial, cervical). It has a large department dedicated to head and neck cancers, as well as an internationally recognized onco-hematology department specializing in the management of multiple myeloma and lymphomas. Additionally, the center has specialists in brain cancers and melanoma and hosts a major department dedicated to early-phase and phase 3 clinical trials.

The study included 36 patients diagnosed with the following conditions:

- Breast cancer: 11
- Ovarian adenocarcinoma: 6
- Cervical cancer: 2
- Squamous cell carcinoma: 5
- Melanoma: 4
- Rhabdomyosarcoma: 2
- Gastrointestinal stromal tumors: 2
- Lung carcinoma: 3
- Mesothelioma: 1
- Medulloblastoma: 1

Our study aimed to include both male and female patients with various pathologies and from different socio-economic backgrounds.

We have incorporated a new table providing a more detailed presentation of the patient cohort. Additionally, we have elaborated on the hypotheses regarding the underrepresentation of men from disadvantaged socio-economic backgrounds in the manuscript. The key points are as follows:

- Patients with head and neck tumors, who are predominantly male and from lower socio-economic backgrounds, are often put into remission following surgery and are not referred to the palliative care department. In cases of recurrence, physicians do not habitually consider palliative care follow-up.
- Patients with brain tumors frequently have a poor prognosis at diagnosis despite maintaining a good general condition. The initial treatment lines (surgery, chemotherapy, radiotherapy) do not significantly impact their overall health, and they are not referred to palliative care. In cases of recurrence, oncologists typically continue patient management until death.
- In onco-hematology, there is limited integration of palliative care. Patients are referred to palliative care at best within the month preceding death, which is incompatible with monthly follow-up via teleconsultation.

Nevertheless, all included patients completed a written questionnaire on their perceptions and expectations regarding teleconsultation before its implementation. We found no correlation between gender, socio-economic status, pathology, or other factors.

Exclusion of 5% of patients due to lack of internet access underlines inequities in teleconsultation availability. Suggest strategies such as providing technical equipment (e.g., tablets, mobile hotspots); establishing community telemedicine hubs for shared access; and offering telephonic consultations as a low-tech alternative. Discuss how these findings could inform policies aimed at reducing the digital divide, such as public-private partnerships to improve digital infrastructure in underserved areas or subsidies for patients to access necessary technology.
Following this study, we established a partnership with the "French League Against Cancer" (a non-profit organization), which has committed to funding tablets with internet access for palliative care patients in need. In the publication text, we propose this partnership model with patient associations or organizations supporting vulnerable populations (non-governmental organizations).

We have also identified an alternative approach to ensure that individuals without digital tools are not excluded from teleconsultations. In France, pharmacies are widely distributed across the entire territory. The vast majority of French citizens have access to a nearby pharmacy, whether in urban or rural areas. The government has funded the installation of teleconsultation booths in all rural pharmacies and a significant number of urban pharmacies. This initiative provides patients with internet access and a dedicated private space for their consultations. These teleconsultation sessions in pharmacies are offered free of charge.

The study briefly mentions educational, cultural, and medical capital but lacks depth on their influence on teleconsultation outcomes. Incorporate specific patient stories or quotes from interviews that highlight how these forms of capital impact engagement and satisfaction with teleconsultations. Provide actionable insights on how teleconsultation practices can be adapted to different social profiles. For instance, patients with lower digital literacy may benefit from tutorials or simplified interfaces, while those with high medical literacy may prefer in-depth discussions.

As we have demonstrated, there is no correlation between the level of education and patients' acceptance of teleconsultations. All included patients (who were not selected based on this criterion, as all had accepted) successfully connected and benefited from the consultation(s). In the discussion, we highlight the importance of caregivers in facilitating the connection. The possibility for patients to conduct their teleconsultation in a pharmacy, with the assistance of on-site staff and free of charge, suggests that teleconsultations could be accessible to everyone, regardless of their educational background.

A total of 48 consultations were conducted, and physicians did not observe any difference in patient behavior compared to in-person visits. We do not believe that the use of a screen for consultations alters patients' understanding. However, it should be noted that our study was conducted with palliative oncology patients. These patients had been followed in oncology for several months or even years, meaning they had developed a certain level of health literacy.

The high mortality rate (64%) within three months of enrolment indicates delayed referrals to palliative care, limiting teleconsultation benefits. Investigate and discuss factors contributing to late referrals, such as healthcare provider hesitancy, lack of patient awareness, or systemic delays. Advocate for integrating palliative care earlier in the oncology care pathway, emphasising benefits beyond end-of-life care. Reflect on how late-stage enrolment may have constrained patients’ ability to adapt to teleconsultations, potentially skewing satisfaction or utility perceptions.

The reviewer is absolutely right. Patients are referred to the palliative care department far too late. As mentioned earlier, this is partly due to the shortage of palliative care physicians (human resources) but, more importantly, to the lack of awareness among oncologists, surgical oncologists, and onco-hematologists regarding the expertise of palliative care specialists. There is a real need for all practitioners to become more familiar with palliative care.

In the collective imagination (both among patients and healthcare providers), palliative care is often associated with death or, at best, with the final days before passing. The current debates in France regarding the potential legalization of euthanasia only reinforce these preconceptions.

French cancer centers, under the impetus of the National Cancer Institute (INCa), have initiated discussions on the early integration of palliative care into the patient care pathway. Our institution will launch joint consultations involving both palliative care specialists and oncologists starting this fall for patients experiencing a recurrence of gynecological cancer (ovarian or cervical cancer). The primary objectives of these joint consultations are to reduce hospitalizations (we hope that this dual follow-up will lead to fewer hospitalization days for patients in the following year) and, most importantly, to prevent the prescription of overly aggressive treatments at the end of life (deprescription). Additionally, this approach is expected to improve patients' quality of life.

If we can demonstrate that dual follow-up enhances these parameters, such consultations will progressively be implemented within our institution and, more broadly, across the 19 French cancer centers.

However, we do not believe that late recruitment has limited patients' ability to adapt to teleconsultations. All patients have adapted very well, and, as mentioned earlier, they have been followed for several months or even years before their admission to the palliative care department. They are already familiar with the institution and the healthcare system.

The absence of standardised quantitative outcome measures weakens the study's conclusions. Include quantitative tools such as patient satisfaction surveys for a standardised view of teleconsultation acceptance, health-related quality of life measures to evaluate broader impacts, and symptom management success rates or reduced hospitalisations. Discuss how these metrics corroborate or challenge qualitative findings, adding rigour to the study’s conclusions.

The acceptability and effective implementation of teleconsultations were assessed using closed-ended questions with limited response options (yes/no). Consequently, the use of standardized satisfaction scales did not seem relevant.

We aim to evaluate the relevance of teleconsultations in palliative care by assessing specific criteria, including medical scales measuring patients' quality of life (mobility, daily activities, nutrition, etc.), caregiver burden, patient anxiety, and depressive symptoms. This will constitute a clinical study involving the collection of precise medical data, adhering to strict regulatory criteria. However, the present study is a social sciences and humanities study. Its primary objective was to identify the typical patient profile that could benefit from monthly follow-up via teleconsultation.

Our findings indicate that all patients benefited from teleconsultations in terms of follow-up satisfaction and that all patients embraced this new mode of care with enthusiasm.

Gendered socialisation is mentioned but not explored in detail regarding its influence on teleconsultation outcomes. Elaborate on how gender roles and expectations may shape teleconsultation engagement. For instance, women may be more accustomed to caregiving roles, influencing their comfort with digital health tools, and communication styles may vary by gender, affecting the dynamics of virtual consultations. Provide evidence or examples from interviews or observations that highlight these gendered differences in teleconsultation experiences.

Indeed, we were only able to assess satisfaction through structured interviews with female patients. Thus, we cannot draw any conclusions regarding gender differences in teleconsultation experiences.

While identifying barriers like digital literacy and connectivity, the study does not propose concrete solutions. Discuss initiatives such as training programs for patients and carers to improve digital literacy and developing user-friendly telehealth platforms with intuitive interfaces. Highlight how healthcare systems and policymakers can address these barriers, including funding for digital tools or legislation to ensure equitable access to telemedicine.

None of the patients included in this study experienced difficulties accessing teleconsultations. The only barrier was financial: two patients lacked digital devices. We have addressed potential solutions earlier.

At the institutional level, the expansion of teleconsultations is hindered by economic factors. A teleconsultation is reimbursed at €23, whereas an in-person consultation is billed at over €120 for the same duration with the patient. As a result, hospital structures are not incentivized to promote teleconsultations on a large scale. A possible solution could involve having junior physicians conduct these teleconsultations while consulting senior physicians in cases of difficulty or increasing remuneration for telemedicine consultations.

The three-month study duration limits insights into the sustained impact of teleconsultations. Suggest follow-up studies spanning six months to a year to assess long-term outcomes, including patient satisfaction, clinical effectiveness, and cost-efficiency. Discuss potential issues in maintaining engagement over time, such as digital fatigue, evolving patient needs, or technological challenges.

The reviewer is correct. However, 64% of patients withdrew from the study before the three-month mark. The 11 patients who completed the study continued receiving monthly teleconsultations, following a schedule of two teleconsultations for every in-person consultation. They all reported being satisfied with this follow-up approach.

Since the study's conclusion, palliative care physicians have continued to offer monthly teleconsultation follow-ups to new patients admitted to the day hospital. We plan to conduct a long-term satisfaction study for these patients. Before initiating the study, we must submit the project for ethical committee review and approval.

6. PLOS authors have the option to publish the peer review history of their article ([what does this mean?](https://track.editorialmanager.com/CL0/https:%2F%2Fjournals.plos.org%2Fplosone%2Fs%2Feditorial-and-peer-review-process%23loc-peer-review-history/1/010f0194952e5f02-9e52ba76-ee60-408f-8c97-8a8e9fee5d8a-000000/YAV7pJa6MIgQt6K2JxH0WHxWURNeQG45RilSvXT9_K0=195)). If published, this will include your full peer review and any attached files.


If you choose “no”, your identity will remain anonymous but your review may still be made public.


**Do you want your identity to be public for this peer review?** For information about this choice, including consent withdrawal, please see our [Privacy Policy](https://track.editorialmanager.com/CL0/https:%2F%2Fwww.plos.org%2Fprivacy-policy/1/010f0194952e5f02-9e52ba76-ee60-408f-8c97-8a8e9fee5d8a-000000/qJYwsQPHiMGfR9oRuoI3l0u6NXeK4TS8-NVQd7N6XH0=195).

Reviewer #1: **Yes:** Andreia Matos

Reviewer #2: **Yes:** Ricardo de Moraes e Soares

[NOTE: If reviewer comments were submitted as an attachment file, they will be attached to this email and accessible via the submission site. Please log into your account, locate the manuscript record, and check for the action link "View Attachments". If this link does not appear, there are no attachment files.]

While revising your submission, please upload your figure files to the Preflight Analysis and Conversion Engine (PACE) digital diagnostic tool, [https://pacev2.apexcovantage.com/](https://track.editorialmanager.com/CL0/https:%2F%2Fpacev2.apexcovantage.com%2F/1/010f0194952e5f02-9e52ba76-ee60-408f-8c97-8a8e9fee5d8a-000000/LvfoIWU1E-gd6g71HleiS66xLy6o3EcaB7_hk1emXco=195). PACE helps ensure that figures meet PLOS requirements. To use PACE, you must first register as a user. Registration is free. Then, login and navigate to the UPLOAD tab, where you will find detailed instructions on how to use the tool. If you encounter any issues or have any questions when using PACE, please email PLOS at [figures@plos.org](mailto:figures@plos.org). Please note that Supporting Information files do not need this step.
