# Supplementary material for: Palliative care follow-up for cancer patients combining day-hospital visits and telemedicine: What feasibility?
Source: PLoS One. 2026 Mar 24;21(3):e0318050. doi: 10.1371/journal.pone.0318050 (PMC13012492; doi:10.1371/journal.pone.0318050)
Supplement: Attachment — Submitted filename: Point-by-point responses to the referees.docx [file pone.0318050.s005.docx]

Pr. Bettina Couderc

CERPOP, UMR 1295, Inserm, Université de Toulouse

Equipe BIOETHICS : "Trajectoires d'innovations en santé : enjeux bioéthiques et sociétaux"

37 allées Jules Guesde

31073 Toulouse cedex, France

[Bettina.couderc@inserm.fr](mailto:Bettina.couderc@inserm.fr)

Toulouse, August 24^th^ 2025,

**Point-by-point responses to the referees**

We thank the referees for the time they devoted to evaluating our manuscript. We are grateful for their constructive feedback, which has allowed us to improve both the presentation and the discussion of our findings.

Below you will find our detailed, point-by-point responses to the reviewers’ comments.

***Referee 1***

*The study provides rich qualitative perspectives; however, it lacks quantitative data that could strengthen its generalizability. A mixed-methods approach including statistical analysis of teleconsultation adherence rates, patient outcomes, and healthcare resource utilization would reinforce the conclusions.*

We acknowledge the many biases in our study. We have expanded the Methods section so that the referees can better assess how we generated the quantitative analyses. The study was conducted in France, where the healthcare system provides free access to care for all. Consequently, adherence to a monthly rather than quarterly follow-up device is 100%. The contribution of teleconsultations to patient medical follow-up is difficult to quantify in a study such as ours with a small sample size. Physicians reported 4 cases out of 38 where teleconsultation provided significant added value (these data are included in the revised manuscript). We have shown that monthly follow-up allows more reactive adjustments in management than if the patient had access to a palliative care physician only every 3 months. We also demonstrate that monthly follow-up improves patients’ perceived quality of life—they feel reassured by monthly specialist follow-up—though we have not quantified this effect.

*Furthermore, the study focuses exclusively on women, which limits applicability to male patients, whose communication styles and engagement in care may differ. Future research should explore gender differences in telemedicine adoption and effectiveness.*

We fully agree with the referee’s observation. Please find our remarks regarding this study bias included in the Discussion. In this study, we included patients without prior selection, based on admission to the department. We included both men and women with various pathologies and from diverse socio-economic backgrounds. Patients with head and neck tumors (predominantly men, often from less favorable socio-economic contexts) are underrepresented because they are frequently put into remission after surgery and rarely referred to the palliative care mobile team. In cases of recurrence, physicians in the department often do not think to propose palliative care follow-up. Patients with brain tumors often have a poor prognosis at diagnosis despite a good general condition. First-line treatments (surgery, chemotherapy, radiotherapy) do not alter their general condition and they are not referred to palliative care. In case of recurrence, oncologists follow these patients until death with only occasional input from palliative care physicians. Finally, in hemato-oncology, there is limited integration of palliative care; patients are referred at best within the month preceding death, making monthly teleconsultation follow-up unfeasible.

*The concept of a “sentinel patient” is relevant but may not apply to all palliative care patients. The study mainly examines patients already familiar with healthcare systems and possessing good health literacy, thereby neglecting populations with lower medical capital, such as those from disadvantaged backgrounds or with limited prior contact with healthcare.*

Our study concerned patients who had been followed for their disease for many years. It is true that they all had good health literacy. We believe that teleconsultations would not be suitable for patients with late-stage disease detected too late (without any specific treatment available), those in precarious situations, or those with a short life expectancy. Teleconsultations are, in our view, best suited for the long-term follow-up of patients already well known to the care teams.

*Moreover, while the study indicates that all patients connected without major technical issues, it does not address those who refused teleconsultations or faced digital barriers. This omission risks providing an overly optimistic view of telemedicine accessibility.*

We have added in the Discussion the possibility for patients to use teleconsultation platforms available in pharmacies (well-established throughout France). These platforms are freely accessible and allow all patients, regardless of social level, to benefit from teleconsultations. They offer broad opening hours nationwide. A study on the use of these platforms in palliative care could be considered.

*The study briefly mentions that older patients showed some hesitation in using digital tools; however, it does not provide an in-depth analysis of technological barriers. Questions remain regarding:
i) Internet availability in rural areas;
ii) The impact of cognitive decline on telemedicine effectiveness;
iii) Platform ergonomics for those with low digital literacy.*

We suggested the use of pharmacy teleconsultation platforms, particularly in rural areas.
Regarding cognitive decline, we believe that teleconsultations are not suitable without a caregiver’s presence.
Pharmacy platforms provide assistance with connecting and, if patients wish, pharmacists or pharmacy assistants can help with understanding the physician’s communication.

*The study highlights the convenience of telemedicine but does not fully address the ethical and emotional implications of remote palliative care. End-of-life care is deeply personal, and face-to-face interactions often provide comfort. The study could have explored:
i) whether patients felt a loss of empathy or emotional connection;
ii) how physicians adapted their communication style to convey empathy through a screen;
iii) the psychological impact of delivering bad news remotely.*

Patients assessed via telemedicine mostly wished to continue such follow-up beyond the study—most alternated in-person and teleconsultation visits, while 2 chose teleconsultations only.

Emotional moments occurred during teleconsultations, sometimes involving both patient and caregiver, often around care and support, loss of autonomy, and approaching death.
Empathy via telemedicine requires time; only vision and hearing are available channels, as touch is not possible. In practice, however, not all patients rely on physical contact for communication.
Regarding breaking bad news, telephone is indeed a poor tool due to the inability to gauge non-verbal reactions; teleconsultations, like in-person visits, allow access to non-verbal cues (only touch is missing, see above).

**Reviewer #3 Comments:**

*Title of manuscript: “Benefits of palliative care follow-up combining day hospital and telemedicine.”*
*We appreciate the opportunity to review this revised manuscript. The topic is highly relevant and addresses an important issue in palliative care: ensuring continuity and accessibility of care despite limited resources. The hybrid model combining day-hospital visits and monthly teleconsultations is aligned with current clinical trends, and the authors have clearly invested significant effort in the intervention and its presentation.
However, major concerns remain regarding scientific structure, methodological transparency, and interpretation of results, which should be rigorously addressed before publication.*

1. ***Structure: Results and Discussion must be separated.***

We have followed the referee’s recommendation by restructuring the manuscript. The Results section is now more factual, while interpretation and reflection are presented separately in the Discussion. A brief concluding section remains. A tracked-changes version is provided for review.

2. ***Methods section lacks detail.***

*The methodological description was previously insufficient for both the quantitative and qualitative components.*

***Quantitative:*** *No information was provided regarding item development, the scales used, their validation, or the analysis plan. Statements such as “no correlation observed” were not substantiated.*

***Qualitative:*** *The thematic analysis was described only briefly, with no details on coding procedures, the individuals involved in coding, whether inter-rater discussions took place, or whether any software was used.*

***Suggestion (as per reviewer):*** *The Methods section should be expanded to offer a clear and reproducible description of both data collection and analysis procedures. Statistical claims should be supported by actual analyses. The Methods should follow a logical structure reflected in the Results.*

We apologize to the referee for the lack of detail in the previous version. We have now carefully expanded the description of the methods, which we hope are now sufficiently clear. The questionnaire and the interview guide translated into English are provided in Annexes 1 and 2.

**Data Analysis**

**Quantitative Analyses:**
Upon inclusion in the study, each patient was assigned an identification number linked to the paper questionnaire they completed. This identification number enabled tracking of the patient’s trajectory (e.g., number of teleconsultations performed) and, when applicable, linking the transcript of the follow-up interview conducted at 3 months. Only the principal investigator (VMS) and the research coordinator (BC) had access to the tables linking patient identities to research-related data.

Questionnaires were analyzed using Excel. The objective was to capture patients’ initial apprehension or confidence toward teleconsultations as well as their knowledge and understanding of the system. Responses were compared according to patient characteristics (age, place of residence, education level, occupational category, etc.). We also compared pre-teleconsultation questionnaire responses with data transcribed from the semi-structured interview for 11 patients. The data were subjected to simple statistical analyses (frequency and mean) and cross-tabulations.

Raw questionnaire data were additionally coded using IRAMUTEQ lexical analysis software (IRAMUTEQ R 3.1.2; free version of ALCESTE) [24]. For each included patient, we recorded gender, age at inclusion, occupational status, education level, place of residence, and the presence of a caregiver. We also extracted certain questionnaire responses (e.g., apprehension or interest regarding teleconsultations [Y/N], need for caregiver assistance to connect [Y/N], etc.). Finally, we added the number of teleconsultations performed and, when available, the corpus of the final interview (IRAMUTEQ allows a distributional statistical analysis to provide significant word occurrences within a corpus).

Item-by-item correlation analyses were performed using Jamovi software [25], which processes IRAMUTEQ outputs. These analyses were verified by the team epidemiologist (SL). Correlation analyses regarding favorable or unfavorable a priori attitudes toward teleconsultations were carried out by combining responses to favorable items (1, 2, 3, 4, 10, 11, 13, and 15) and unfavorable items (5, 6, 7, 8, 9, 12, 14, and 16).

**Qualitative Analyses:**
We conducted a sociological thematic analysis of the 11 interviews [26], as well as an IRAMUTEQ-based lexical analysis. The IRAMUTEQ analysis of interview transcripts did not identify any significant text clusters. Regarding the sociological thematic analysis, the method used was manual and theme-based, consisting of transposing the corpus into a set of representative themes related to the research question. The corpus was analyzed by the research team, which included a sociologist (AFR), an ethicist (BC), the head of the institute’s palliative care department (NCH), and the physicians (VMS, MB). The thematic sociological analysis was produced through the triangulated perspectives of the study authors. The thematic analysis thus combined the quantitative outputs from IRAMUTEQ/Jamovi with an interpretive analysis of the meaning conveyed in participants’ responses. No additional software was used.

3. ***The title overstates the scope of the study*** *:The current title suggests that “benefits” have been demonstrated as a result of the hybrid model. In reality, this is a short-term feasibility and acceptability study, based on a small sample and descriptive data, without any comparative evaluation of outcomes.*
   ***Suggestion:*** *Revise the title to better reflect the actual scope of the study, for example: “Feasibility of a hybrid follow-up model combining day-hospital care and teleconsultation in palliative oncology” or an equivalent formulation.*
4. We propose a new title consistent with the reviewer’s suggestion: *“Palliative care follow-up for cancer patients combining day-hospital visits and telemedicine: what feasibility?”*

***4. Interpretation must be more nuanced*** *The manuscript sometimes draws conclusions that are not directly supported by the data. For example, statements regarding symptom management or the reduction of emergency admissions remain hypothetical and should be presented as such. Similarly, the interesting sociological concept of the “sentinel patient” should be articulated with greater caution, given the absence of comparisons between patient groups.*
***Suggestion:*** *Rephrase these interpretations as hypotheses or avenues for future research, rather than demonstrated effects.*

We are fully aware that our small sample does not allow for generalization, and we have revised the discussion accordingly. We have also provided a more detailed description of four patients who clearly derived measurable benefit from teleconsultations in the Results section:

“Some teleconsultations allowed the improvement of distressing symptoms: for two patients who experienced increased pain, management consisted of an adjustment of analgesic treatment for one and the initiation of a WHO step III analgesic for the other. The advantage of these two teleconsultations compared to a simple telephone call was that they allowed us to observe how the patient moved and provided information relevant to the etiological diagnosis (despite the absence of a physical examination). A prescription was then sent to the pharmacy and to the patient.
For another patient, in light of a deteriorating general condition and difficulties maintaining care at home, the initiation of hospital-at-home (HAD) services was proposed: this patient was able to die at home without transfer to the emergency department or inpatient ward (contrary to her wishes). For another patient, a systematic teleconsultation allowed the identification of a femoral fracture: she underwent plain radiography confirming the fracture; her case was then directly discussed in the pain and bone metastasis multidisciplinary tumor board, and she was admitted directly to orthopedic surgery, bypassing the emergency department, which would have been ill-suited in this palliative context. Finally, for another patient, visualization of an exudative tumor wound during the teleconsultation, in collaboration with the home-care nurse present, allowed adaptation of the care protocol and thus improved the patient’s comfort.
In the absence of these tailored interventions or treatment modifications, these patients would likely have required hospitalization, or even ended up in the emergency department—settings often inappropriate in the palliative context and not necessarily aligned with patient wishes.
Moreover, in oncology, due to the long medical trajectory, patients often “wait” until the situation has severely deteriorated before seeking care through emergency services, even when this is not optimal. It should be noted that for the patients described above in our study, the systematic use of teleconsultations helped identify symptoms, even though patients had not alerted the mobile palliative care team despite having their contact information. Each issue was addressed and contributed to improving their quality of life. However, we acknowledge that, given our small sample size, these findings can only be considered exploratory and represent directions for future research.”

***5. The qualitative report should be more focused*** *The inclusion of patient quotations is valuable; however, some excerpts are lengthy and redundant, which undermines the readability and clarity of the thematic presentation.*
***Suggestion:*** *Shorten representative quotations where possible, and consider providing a summary table of themes and illustrative excerpts to improve structure and clarity.*
We have revised the quotations to make them more concise and targeted.

**6. The limitations regarding generalizability should be further emphasized**
*The sample is small, predominantly composed of relatively advantaged women, and drawn from a single institution. While these limitations are mentioned, they should be discussed more explicitly in relation to the feasibility of broader implementation. The study population is likely highly selected (e.g., access to digital tools, caregiver support, familiarity with the healthcare system).*
We have tempered our statements accordingly to align with the reviewer’s comments.

**Conclusion**
*This is a valuable and well-motivated pilot study, and we commend the authors’ commitment to improving healthcare delivery. However, substantial revisions are required to meet core standards of scientific communication. I recommend a major revision, including a clear restructuring of the manuscript and strengthening of both methods and interpretation. I am willing to review a revised version that addresses these points.*

We are pleased to submit a revised version incorporating a comprehensive set of corrections addressing the comments from both reviewers. Please find enclosed the final version as well as a tracked-changes version to facilitate your assessment of the revisions.
We sincerely hope that our responses meet the reviewers’ expectations.
