# Supplementary material for: Palliative care follow-up for cancer patients combining day-hospital visits and telemedicine: What feasibility?
Source: PLoS One. 2026 Mar 24;21(3):e0318050. doi: 10.1371/journal.pone.0318050 (PMC13012492; doi:10.1371/journal.pone.0318050)
Supplement: Attachment — Submitted filename: Point-by-point responses to the referees132.docx [file pone.0318050.s006.docx]

Pr. Bettina Couderc

CERPOP, UMR 1295, Inserm, Université de Toulouse

Equipe BIOETHICS : "Trajectoires d'innovations en santé : enjeux bioéthiques et sociétaux"

37 allées Jules Guesde

31073 Toulouse cedex, France

[Bettina.couderc@inserm.fr](mailto:Bettina.couderc@inserm.fr)

Toulouse, February 13^th^ 2026,

**Point-by-point responses to the referees**

We thank the referees for the time they devoted to evaluating our manuscript. We are grateful for their constructive feedback, which has allowed us to improve both the presentation and the discussion of our findings.

Below you will find our detailed, point-by-point responses to the reviewer N°4 comments.

***Referee 4***

*The methodology is described in a valid manner. The text states that a feasibility assessment model was adopted. However, the adopted model was not aligned with the assumptions for conducting feasibility studies. The text is silent regarding the justification of the recruitment rate, retention parameters, acceptance thresholds, and feasibility criteria. The feasibility analysis is retrospective and descriptive. The situation limits the validity of the analysis and hinders the evaluation of the model's application. It is suggested that the feasibility outcomes be defined a priori, such as the completion rates of admissible teleconsultations, the proportion of eligible patients, and the acceptable rates of technical failures. It is also suggested that inferences regarding feasibility be related to predefined benchmarks, and in case of impossibility, the limitations should be acknowledged and that feasibility was assessed in an exploratory rather than systematic manner.*

To evaluate the feasibility of teleconsultations among all patients with access to a connected device, we deliberately applied no additional selection criteria beyond a WHO performance status ≥ 3. Age, accompaniment status, and educational level were not considered exclusion criteria.

We have added two sentences to the Methods section clarifying how acceptability and feasibility were assessed. Acceptability corresponded to the percentage of patients with connected devices who agreed to participate in the protocol when it was proposed to them (100%). Feasibility corresponded to the percentage of patients who were able to successfully connect to their medical consultations (48 teleconsultations completed out of 48 scheduled, including two that were postponed by a few days due to holidays or poor internet connectivity).

Our aim was to demonstrate that teleconsultation could be successfully implemented regardless of patients’ age or digital literacy. All included patients were able to connect and complete their teleconsultation, supporting the feasibility of this approach provided that access to a connected device is ensured.

In the Discussion section, we outline potential solutions for patients lacking access to connected devices. Given the reliability of the internet infrastructure in France, major technical failures were not anticipated. In cases of network or device malfunction, appointments could be rescheduled, as would be done for in-person visits. This occurred in 2 of the 48 consultations conducted.

*The inclusion criterion, estimated life expectancy ≥3 months, is not operationalised or justified, and the imprecision is evidenced by the high mortality rate (64%). The situation raises concerns about the validity of the selection and adequacy of the eligibility assessment, which affects the interpretation of the results. It is suggested that the clinical criteria for estimating life expectancy be described. It is also suggested to discuss the implications of inaccurate prognostication on the validity of the study. The limitations and biases should be discussed, and the sample reformulated.*

To date, medicine does not provide reliable scientific tools to accurately quantify the number of weeks or months of remaining life expectancy. Moreover, in this palliative and symptom-oriented context, and considering the benefit–risk balance within an ethical framework, we chose not to perform additional biological investigations and did not use biological parameters as selection criteria.

Inclusion in the study was based on clinical criteria only: absence of major vital organ failure (hepatic, pulmonary, or cerebral involvement), a WHO performance status ≤ 3, and the clinical judgment of the disease-specific oncologist referring patients to the palliative care department, who estimated a life expectancy greater than three months.

These characteristics have been added to the Materials and Methods section.

The primary objective of this exploratory study was to assess the feasibility and acceptability, among patients receiving palliative cancer care, of monthly remote follow-up (teleconsultation) with a physician they had met only once (i.e., without an established therapeutic relationship), with the aim of implementing this model sustainably in comprehensive cancer centers.

The potential clinical impact in terms of preventing emergency department admissions through monthly rather than quarterly follow-up is mentioned in the manuscript as an observational finding only. Given the small sample size, no statistically robust conclusion could be drawn. This was not the primary objective of the study, as no longitudinal clinical outcome data were collected during follow-up. The manuscript has been revised (abstract and main text) to clarify that clinical benefit was not the primary endpoint, particularly due to the absence of predefined evaluation criteria.

Although a high mortality rate was observed, this does not affect the validity of our feasibility study, which was not designed as a quantitative outcome study. Mortality limited the number of final interviews that could be conducted. Nevertheless, the total number of teleconsultations performed (n = 48) and the number of completed final interviews (n = 11) consistently support the feasibility of teleconsultation, even in a palliative oncology setting.

Teleconsultation was perceived as beneficial by patients, who reported that monthly follow-up was more appropriate and reassuring than a single quarterly in-person visit. The qualitative interviews were consistent, and data saturation was achieved.

To evaluate feasibility without focusing on a specific patient subgroup, participants were included consecutively as they were referred to the department. Patients with prior contact with the service (i.e., with an existing physician–patient relationship) were excluded in order to avoid bias related to a pre-established therapeutic bond.

*Quantitative methods depend on specific items from the questionnaire, which have not been validated or subjected to psychometric evaluations. The text is also silent regarding the reliability, construct validity, or consistency of the composite indices.*

Participants completed a self-administered questionnaire. The instrument included various types of items: single- and multiple-choice questions, as well as 5-point Likert-type scales. Likert scales were used to assess the degree of agreement with specific statements (e.g., ranging from “strongly disagree” to “strongly agree”). These formats were selected to better capture patients’ opinions, perceptions, and preferences, while remaining brief and easy to complete for patients who were often fatigued.

The survey focused on several key domains:

- General perceptions of telemedicine (feasibility)
- Perceived personal relevance and adequacy of telemedicine within their individual care trajectory
- Relationships with medical specialists
- Relationships with relatives

These domains were intended to explore the human and social dimensions of patients’ perspectives on this model of follow-up.

We have elaborated on this point in the Materials and Methods section, as suggested by the reviewer.

The questionnaire was developed collaboratively by all co-authors of the publication (physicians, a sociologist, statisticians, and ethicists). It was reviewed and approved by the Research Ethics Committee of the Federal University, which includes four psychologists among its members. The committee formally accredited the study and validated the methodological framework as part of its area of expertise (IRB number assigned).

*The conclusions about the patients' behaviours, reassurance, and perceived benefits are based on measures of uncertain validity. It is suggested to describe the assumptions behind the construction of the questionnaire or, alternatively, to acknowledge the lack of validation, which represents a significant methodological limitation. It is also suggested to exercise more moderation in interpreting the questionnaire results and that the conclusions be limited solely to the observed evidence.*

We have revised the manuscript to adopt a more cautious tone, as suggested by the reviewer. Specifically, we have moderated our statements by clearly indicating that the study is qualitative, descriptive rather than aiming at providing a quantitative evaluation. This study is exploratory, descriptive, and qualitative, and was not designed to provide quantitative conclusions.

*The statistical analyses are insufficient to support the inferences of absence of associations between demographic variables and the feasibility or acceptability of teleconsultation. Due to the sample size, skewed distributions, and limited statistical power, non-significant results cannot be interpreted as evidence of the absence of effect.*

We did not state that there was no effect but only that “No correlation **was observed** between genre, residence, education or profession and attitudes teleconsultation”. We completely agree with the reviewer on the fact that sample size did not allow for inferences and that is why we did not adopt a quantitative reading of the results. As we stated in the discussion, in the recruitment and sample characteristics section “This study included a small sample of 36 patients. Therefore, the quantitative descriptive analyses should only be considered as illustrative of the results of the qualitative analyses, which were the core of this work.”

*It is suggested to reformulate the text. It is also suggested to replace definitive terms (did not influence) with more probabilistic terms (no statistically detected association in the sample), acknowledging the risk of Type II error. The text should avoid conclusions related to equity that are not supported by the obtained results. Qualitative analysis lacks methodological rigour to support conceptual interpretations.*

We have revised the manuscript in accordance with the reviewer’s comments. We have also removed the conclusion regarding equity from the abstract, as suggested.

*The description of the results suggests the existence of beneficial contributions from teleconsultation. However, the data only allow us to state that teleconsultation enabled clinical interventions in individual cases but do not allow us to conclude that it caused improvements in clinical outcomes or that it prevented hospitalizations. Given the absence of comparison terms, the interpretations of the results exceed the evidentiary strength of the data. It is suggested to reclassify the examples as illustrations of descriptive cases rather than observed evidence. The interpretations have a hypothetical nature and are speculative.*

We agree with the reviewer and, as mentioned above, have revised the manuscript accordingly. *There is an observed confusion between feasibility, acceptability, and perceived benefit throughout the Results and Discussion sections. Although patient satisfaction and positive perceptions have been documented, the results do not equate to feasibility in the target population, especially considering the exclusion of patients without digital access and those who passed away early. It is suggested that feasibility (implementation capacity), acceptability (patient perception), and perceived benefit (subjective value) be described as distinct constructs with their respective limitations separated. It is also suggested that evidence be emphasized.*

We have reorganized the manuscript by separating the sections addressing feasibility, acceptability, and benefits, in accordance with the reviewer’s recommendations.

We are pleased to submit a revised version incorporating a comprehensive set of corrections addressing the comments from both reviewers. Please find enclosed the final version as well as a tracked-changes version to facilitate your assessment of the revisions.
We sincerely hope that our responses meet the reviewers’ expectations.
